# Biodegradable Binary and Ternary Complexes from Renewable Raw Materials

**DOI:** 10.3390/polym13172925

**Published:** 2021-08-30

**Authors:** Agnieszka Folentarska, Jakub Łagiewka, Magdalena Krystyjan, Wojciech Ciesielski

**Affiliations:** 1 Faculty of Exact, Natural and Technical Sciences, Jan Dlugosz University in Czestochowa, 13/15 Armii Krajowej Ave., 42-200 Czestochowa, Poland; a.folentarska@ujd.edu.pl (A.F.); j.lagiewka17@gmail.com (J.Ł.); 2 Faculty of Food Technology, University of Agriculture in Krakow, 122 Balicka Street, 30-149 Krakow, Poland; magdalena.krystyjan@urk.edu.pl

**Keywords:** polysaccharides, proteins, fats, blends, complexes

## Abstract

The aim of this paper is to investigate the interactions between polysaccharides with different electrical charges (anionic and neutral starches) and proteins and fats in food ingredients. Another objective is to understand the mechanisms of these systems and the interdependence between their properties and intermolecular interactions. At present, there are not many studies on ternary blends composed of natural food polymers: polysaccharides of different electrical charge (anionic and neutral starches), proteins and lipids. Additionally, there are no reports concerning what type of interactions between polysaccharide, proteins and lipids exist simultaneously when the components are mixed in different orders. This paper intends to fill this gap. It also presents the application of natural biopolymers in the food and non-food industries.

## 1. Introduction

In terms of political (e.g., raw material dictates), economic and ecological (e.g., environmental pollution, particularly during chemical coal processing) factors, and considering the depletion of petrochemical and carbochemical raw materials, increasing attention has been paid to natural renewable raw materials, e.g., polysaccharides, proteins and lipids. Biodegradable materials, such as wood, animal skin and intestines, have been utilised since ancient times. Reed and straw, wool and cotton and, in the very earliest times, papyrus, parchment and paper, were all in common use. The natural rubber (which has been known of since the late Middle Ages) boom began after the development of sulphur treatment by Goodyear (vulcanization). Goodyear developed rubber that was appropriate for, among other things, tires and inner tubes. Rubber, heavily treated with sulphur, was known as ebonite. However, the biodegradability of these products was low. At the turn of the century, Henry Ford boasted of manufacturing a suit made of fibre obtained via formaldehyde treatment of a protein. In terms of biodegradable plastics, galalith (obtained from acid casein) [1] and other products of weakly nitrated cellulose—cellophane and celluloid—became widely popular.

The usability of biodegradable plastics is determined from their physical properties such as mechanical and thermal resistance, barrier properties, their adequate decomposition time in the environment and resulting decomposition products. None of the above listed plastics were characterized by properties that would be satisfactory from this point of view, and their viability does not even take their production costs into account.

In the 1980s, attempts were made to introduce single-use products, such as starch mouldings and extrudates, which were used for the production of, for instance, crockery, moulds and packaging [2,3,4,5,6,7]. However, their mechanical resistance and durability were too low; therefore, a proportion of natural fibres [8] and synthetic polymers [9] were added to the starch. The combination of starch with synthetic, though biodegradable, polymers, such as polyesters, resulted in slowly decomposing type Mater-Bi polymers [10,11]. Considerable popularity was gained by biodegradable polymers obtained without the use of starch. These included poly[lactic] acid and polylactides [12,13,14,15,16,17] and other poly[hydroxyalcanoic acids] [18,19], as well as their copolymers with low and high molecular molecules [20,21,22,23]. Biodegradable synthetic plastics include polyester (poly[caprolactone]) [24,25] and polyamide (poly[caprolactame]), also known as nylon 6 [26].

At the same time, so-called green plastics were starting to be used, such as complexes produced from starch (up to 40%) moulded into vinyl polymers, for instance, polyethylene. However, such plastics were better geared to the requirements of aesthetics rather than ecology. Starch decomposes into CO_2_ and water reasonably quickly, leaving in the environment a synthetic polymer that is undecomposed, yet dispersed and invisible to the naked eye [27,28].

Synthetic plastics that decompose entirely to CO_2_ and H_2_O and are made of polymerized vinyl monomers, containing appropriate catalysers (d2w plastics) are known, but, due to their origin, they do not fulfil the EU directive on the use of renewable materials. Moreover, their production requires the use of an excessive amount of energy [29]. However, such requirements can be met by complexes produced from polysaccharides, proteins and lipids.

Macronutrients are chemical compounds responsible for providing the human body with energy; macronutrients are grouped as saccharides, lipids and proteins. These compounds are consumed in large amounts in comparison to vitamins or minerals, and also occur together in many food systems. Food processing may modify the properties and interactions of macronutrients, for instance, in terms of flavour, taste, texture, shelf life or nutritional value [30,31]. The interactions are very complex, especially for binary and ternary systems; understanding their occurrence during food processing may be useful in optimized the production and development of new food products with designed microstructures and functionality [32,33].

Polysaccharides: starch, carboxymethylcellulose, pectins, synthetic phosphorylated starch, carrageenans, hyaluronic acid, xanthan gum.

Lipids: esters of stearic, oleic acid.

Proteins: albumin, lysozyme.

The binary systems from natural biopolymers (polysaccharides, lipids or proteins) have been extensively studied, and the binary interaction effects of their properties have been characterized. The formation of starch-lipid complexes exhibits the reduced swelling and solubility of starch, as well as there being retarded gelatinization and retrogradation, and the enzymic digestion rate is slowed down [34,35,36,37]. There are differing possibilities for the formation of binary and ternary complexes due to changes in the mixing order of compounds [32].

Binary complexes have been the subject of intensive research for 50 years; however, the ternary complexes between starch-lipid-proteins have become a new field of investigation with an increasing number of discoveries and applications. This review introduces information concerning ternary complexes, interactions, preparation methods, analytical techniques and applications in the food industry.

## 2. Starch-Lipid Interactions

**Figure 1:**
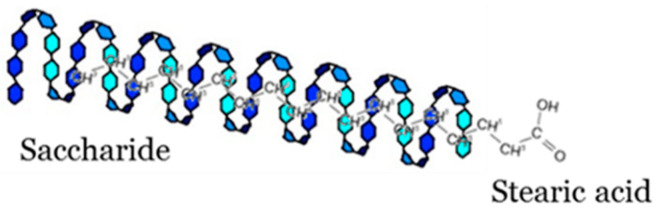
Proposed structure of saccharide-stearic acid.

Polysaccharides form helical complexes with lipids [38] (Figure 1). Formation of a helix is possible thanks to the suitable orientation of the polysaccharide chain in relation to the hydrophobic lipid thread. Due to this, the external portion of the complex becomes hydrophilic. Such ordering of the structure is most commonly used for the implementation of the appropriate texture and thixotropic properties of foodstuffs (doughs, creams, sauces) and cosmetics.

The main driving force to form a complex between starch and lipids is by inclusion complexation naturally in native starch or during heating-cooling protocols. Inclusion complexation involves a series of non-covalent interaction: hydrogens bonds, hydrophobic attractions, van der Waals forces [39,40].

The outer surface contains the hydrophilic hydroxyl groups of α-1,4 glucan helices; additionally, methylene groups and glycosidic bonds line the inner core and form a hydrophobic cavity with an ability to accommodate proper ligands. The glucan sites with the presence of lipids are composed of six glucosyl residues per turn; however, there are seven or eight glucosyl units per turn that have other ligand types. The amylose-lipid complexes can form partially ordered structure known as a V-type crystalline form, which can be grouped as anhydrous V_a_ and hydrated V_h_ forms [34,41,42]. Structural research has indicated the insertion of lipids into the cavity of the amylose helix. The carboxyl group of fatty acids (FAs) or the glyceride moiety are influenced by steric hindrance and electrostatic repulsions; thus, the hydrophylic group is directed to the outside of the helix. Further self-assembling forms crystalline lamelae for the lipid containing helices, where the helices are oriented perpendicularly to the plane of the lamelle. There is a theory concerning micron-sized spherulites, which are formed by the crystalline lamelle and the interspacing amorphous regions. On the other hand, the lipids have weaker binding with highly branched amylopectin in comparison to amylose. Furthermore, several studies exist that evidence the interaction of amylopectin-lipids, but few studies indicate an ability to order crystalline complexes [43,44].

## 3. Starch-Protein Interactions

**Figure 2:**
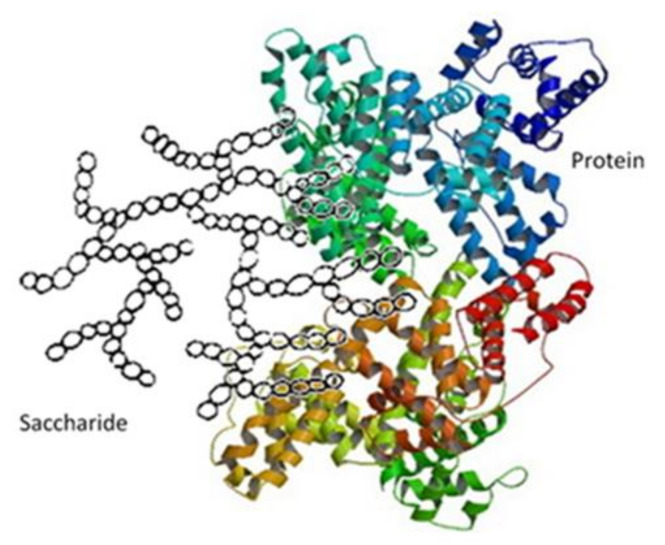
Proposed structure of saccharide-protein.

In the literature, one can find articles [45,46,47,48,49,50,51,52,53,54,55,56,57,58,59,60,61,62,63,64,65,66] on the synthesis of binary polysaccharide-protein complexes. The polysaccharide-protein complexes were obtained either via electrosynthesis or precipitation at the isoelectric point. Some of these complexes are characterized by the resistance of styrene-butadiene copolymers (Figure 2).

Starch–protein interactions involve forces such as hydrogen bonds and electrostatic and van der Waals forces. The hydrogen bond’s ability is due to a high level of donor and acceptor-like hydroxyl groups in starch and nitrogen and oxygen atoms in protein. Electrostatic interactions are formed between oxygen/hydroxyl groups and mostly ionized carboxylate, the ammonia groups of side chains and amide bonds [67,68]. Due to weak ionization of oxygen in starch, the electrostatic force is not always the main driving force.

For instance, potato starch contains additional phosphate groups which interact strongly with wheat protein positive charges and adsorb wheat protein and convert it to starch. The additional protein in starchy products with different pH allows them to modulate the water binding capacity and reduce the viscosity of products. The binary complex increases gel strength due to increased protein density [69]. For starch–gluten systems, a surface interaction between granuls and the gluten molecules can be observed. An increase of storage modulus during the addition of gluten to wheat and rye starches indicates the possibility to facilitate the granule–granule contact and to enhance formation of a transient network [70]. Other studies relating to starch–sodium caseinate have shown behaviour as a power-law fluid with shear thinning and increased viscosity. The increased viscosity may have an influence on the swelling volume; furthermore, swollen granules collapse due to pasting and diffusion of starch from the gelatinized granule to the bulk solution. This means the system may be limited by the protein continuous phase; thus, the net effect is responsible for increasing the volume by limiting disintegration [71]. The starchy products have different gel properties with different media pH. The water binding capacity increases with increased pH for lower protein solubility, which is the same as its relation with viscosity. However, an inverted relation is present for starch–albumin gels with media pH. The gel microstructure is depended on the pH, but the microstructure differences does not provide hardness changes [72,73].

## 4. Lipid-Protein Interactions

**Figure 3:**
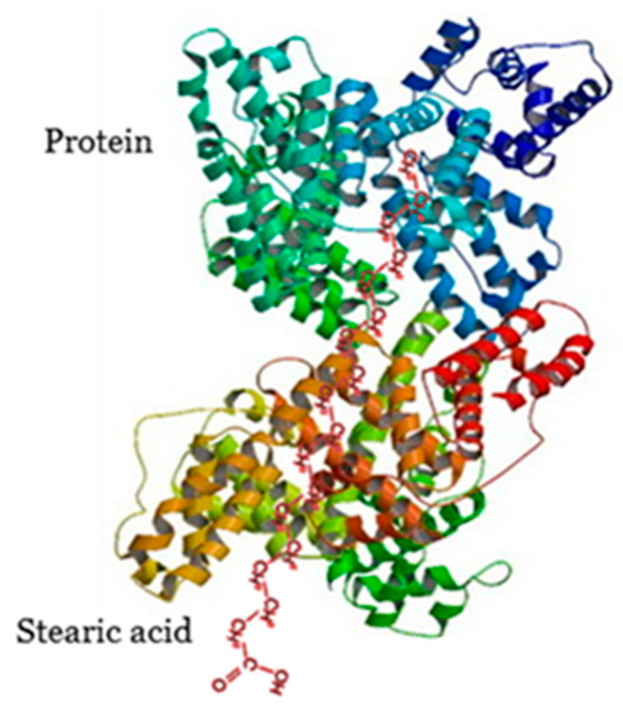
Proposed structure of stearic acid-protein.

Lipid-protein complexes (lipoproteins) are interesting, primarily because of their physiological importance (Figure 3). Research has focused on the interaction of cellular membrane proteins with lipids [74,75,76,77,78], which is linked to the functioning of these membranes, as well as intracellular and extracellular lipid behaviour. Such types of interaction create opportunities to recreate cell membranes [79]. Due to their elevated affinity to lipids, lipoproteins are responsible for the transport of lipid and lipid-like substances within organisms [80]. The structure of cell membranes has been used for the construction of osmotic and dialysis membranes [81].

Lipid-protein binary complexes are formed via the hydrophobic affinity of long-chain FAs to the binding pockets, which contain mostly alkyl and aromatic aminoacids. The binding pockets are obtained from the folding of protein chains due to secondary, tertiary and quaternary structural factors. The conformational factors are driven by van der Waals forces, which favour aggregation, but the carboxylic group as a polar moiety is found to be on the outer part of biomolecules. The carboxylic group is always found around the non-anionic group due to conflicts of electrostatic interactions; the polar group may freely interact with water molecules [82,83].

There appears to be protection of the long aliphatic chain of FA via its hydrophobic affinity inside macromolecules. The protein can complex FAs in different stoichiometries, such as human α-lactalbumin (α-LA). The FA-α-LA complex is characterized by a slight reduction of secondary structure and total loss of tertiary structure due to a fluctuating structure with strongly reduced stability [84]. Another example of conformational changes is for the FA-human serum albumin, which loses tertiary structure in the same manner as the previous system. The conformational changes were only observed in the crystal structure, but there is no evidence for identical conformation in solution [85].

## 5. Starch-Lipid-Protein Interactions

The standard procedure of the ternary complex leads to inclusion of lipids into starch and then the adherence of protein onto the starch surface. The lipid forms inclusion with the alkyl chain, and then the carboxyl group of FA and hydroxyl of starch interact with protein [34]. For instance, β-lactoglobulin and whey protein possess an isoelectric point under a pH value of 6; these proteins can be used as model biomolecules in ternary complexes. Under the isoelectric point of protein, carboxyl groups of FA and positively charged proteins show electrostatic interaction. The main problem is with the pH of starchy foods, which have almost neutral pH and may form anion–anion interactions with proteins and FA [86]. Another protein, type-A gelatin, is acidly-hydrolyzed from collagen, and the isoelectric point value found is 8.0–9.0. Thus, A-type gelatin should be positively charged at a neutral medium, and anion–anion interactions do not collide [87]. On the other hand, shorter alkyl chains and a lower degree of unsaturation favour ternary complexes, but are less thermally stable [88]. Ternary complexes may exhibit a V-type XRD pattern, which is present in starch–FA complexes. Ternary systems are characterized by a greater amount of long- and short-range structural order compared to the binary systems [89].

## 6. Preparation Methods of Binary and Ternary Complexes

**Figure 4:**
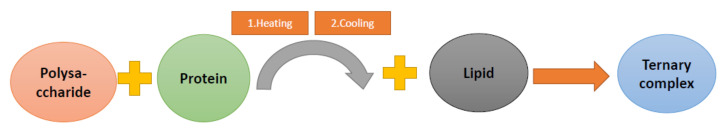
Thermomechanical method for preparation of ternary complex in a Rapid Visco Analyzer (RVA).

There are several different methods for obtaining ternary complexes of starch-lipid-protein, which may differ with stoichiometry, order of added compound or type of ingredients [34,90]. Despite a lack of enzymatic methods, there are some known standard procedures:

- Classical method. The starch solution is heated in a boiling water bath and is then cooled overnight at room temperature. A water solution of protein and FA are added to the aqueous starch solution, which is then heated in a boiling water bath, kept overnight at room temperature and is finally centrifuged. The supernatant consists of starch–lipid–protein complexes. The method is based on a standard protocol based mostly on the preparation of binary complexes [91].
- Thermomechanical method. The ternary complex is produced in a Rapid Visco Analyzer (RVA) by adding starch, protein, lipids and water. The obtained complex is then frozen in liquid nitrogen, freeze-dried and then ground. The obtained powders consist of starch–lipid–protein complexes [92] (Figure 4).
- Enzymatical method. Ternary complex may be obtained via two strategies. The first method is fully enzymatic and focuses on polymerization of the primer to branched biopolymers. The second method is based on enzymatic hydrolysis of the branched biopolymer into smaller parts, which can then interact with other biopolymers. It can then be formed into binary complexes [91,92].

## 7. Edible Biodegradable Films

A ternary complex can be formed from biopolymers, which are natural polymers with biodegradability. Furthermore, the biopolymers can be the edible compounds of food. Nowadays, biopolymers are used to form films for food coating, but there is still a necessity for edible types. The edible films are mostly formed from one or two compounds; nevertheless, three-component systems have become promising film systems. Based on structure, compounds and preparation, the ternary complexes exhibit different bioproperties [93,94].

**Figure 5:**
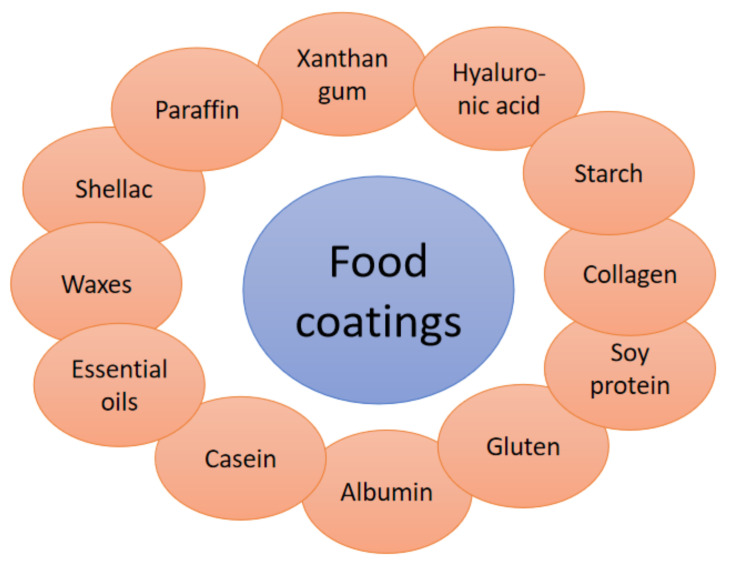
Most popular biopolymers in food coating.

Most typical biopolymers and properties in food coating (Figure 5):

- Edible films from starch are formed mostly by high-amylose starches (containing at least 70% amylose). The starch films are characterized by good elasticity and oxygen impermeability, as well as oil/fat resistance or solubility in hot/cold water. Moreover, the starch packaging exhibits an ability to bind water molecules from products and then decrease the activity of water in food with the reduced development of pathogenic microorganisms. Thus, the starch films make good coatings for bakery products and can increase the freshness of meats or other foods such as fruits and vegetables after freezing [95,96,97,98].
- The most popular type of proteins applied in food coatings are collagen, gelatin, casein, soy protein, gluten or albumin protein. Collagen coatings are mostly used in meat packaging and are characterized by insolubility and reduced loss of meat juice during heating, and they also blend well with meat. The next protein, gelatin, is mostly applied in the microencapsulation of food flavours. Furthermore, the gelatin’s coating allows the limitation of water evaporation from meat and the development of bacterial microflora, and can migrate fatty substances. Casein, as a dairy protein, is characterized by good mechanical properties, good solubility in water and the improved nutritional value of food. Dairy proteins’ films are used to cover fruits, vegetables, dairy products and even meat products [98,99,100,101,102].
- Films made only from lipids are extra brittle and thicker due to hydrophobicity. On the other hand, films based on the addition of lipids are one of the most mechanically stable, and they have barrier residences and prevent moisture migration. Thus, the binary or ternary complexes with lipids are the best solutions, rather than simple lipid films. From lipid binary or ternary blends, there are found a wide range of products such as essential oils, waxes, paraffin, acetyloglycerides and shellac. Those coatings are preferable for covering meats and sometimes citrus vegetables [103,104,105].

## 8. Applications of Renewable Raw Materials

### 8.1. Food Industry

Over 40% of the total demand for plastics comes from the food industry. Although conventional packaging has many advantages, one disadvantage—the lack of biodegradability—means that they pose a high risk to the natural environment and, consequently, to human health [106]. One alternative to plastics are natural, biodegradable polymer complexes, which are successfully used in the food industry, mainly as an element of packaging—a tool that allows one to maintain high quality and the safety of food products. La Mantia et al. proved that biodegradable systems can successfully compete with traditional, non-biodegradable polyethylene-based blends [107]. The results showed that PLA/PBAT (poly(butylene-adipate-co-terephthalate) and poly(lactic acid)), as well as MaterBi, i.e., an extrusion grade with proprietary composition, based on biodegradable aliphatic andaliphatic/aromatic polyesters, showed good potential as a biodegradable polymer systems for agricultural product packaging, especially for the production of nets for the packaging of fruits and vegetables.

#### 8.1.1. Nanomaterials

The common packaging polymers used in the food industry are starch, chitosan, cellulose and alginates [108,109]. Starch films show poor mechanical and low barrier properties; hence, starch has been complexed with other polymers, thereby improving the physical and mechanical properties of such films. For example, Krystyjan et al. reinforced starch-based films with psyllium mucilage in order to obtain natural, edible and biodegradable films with improved mechanical and functional properties [110]. Kasmuri and Zait used eggshell and chitosan as fillers in potato starch to overcome the inherent drawbacks of bio-plastic [111]. Unfortunately, despite this, natural polymers still have many shortcomings. Low thermal stability, high moisture absorption and poor mechanical strength are all particularly problematic [106]. Introducing an added component in the form of a nanomaterial can strengthen the structure of the entire complex and thus allow new, still biodegradable materials with greater efficiency to be obtaining. Nanocomposites consist of a polymer matrix as a continuous phase and nanomaterials as a discontinuous phase, with dimensions in the range of 1–100 nm [112]. Lee et al. proposed active nanocomposite films with antimicrobial activity. They incorporated silver nanoparticles (AgNPs) into pectin/pullulan complexes. According to obtained data, silver nanoparticles improved the mechanical properties of pullulan/AgNPs and pullulan/AgNPs/pectin composites and showed high antimicrobial activity against food borne pathogens: *Salmonella Typhimurium*, *Escherichia coli* and *Listeria monocytogenes*. One of the most commonly used nanomaterials are carbon-based materials. Graphene and other graphene family nanomaterials play one of the leading roles in the development of nanotechnology due to their unique properties [113]. Krystyjan et al. describes a green synthesis preparation of bionanocomposites consisting of starch/chitosan/graphene oxide (GO). The authors claimed that the tensile strength of composites with GO nanoparticles were comparable with commodity plastic films such as HDPE (*High Density Polyethylene*) and LDPE (*Low Density Polyethylene)*. Additionally, cell-based analyses showed no toxic effect of the composites on HaCat keratinocytes and HepG2 hepatoma cells [110]. Jamróz et al. developed films based on furcellaran (FUR) and nanofillers (graphene oxide (GO), multi-walled carbon nanotubes (MWCNTs) and silver nanoparticles (AgNPs)) via a solution casting method. Nanocomposite films with AgNPs showed antimicrobial activity against pathogenic bacteria and fungi (*Pseudomonas aeruginosa*, *Enterococcus faecalis and Staphylococcus aureus*) [114].

#### 8.1.2. Antimicrobial Materials

As a result of changes in the food preferences of consumers and trends in industrial production, new food packaging technologies are developing. These extend the shelf life, maintain the safety and control the quality of food [115]. One of the many ways to extend the shelf life of food is to use packaging polymers with biological properties, e.g., antibacterial and antifungal activity. Antimicrobial properties of packaging result from the type of polymer material, which contain bound or leaching antimicrobials. They may also exhibit varied mechanisms: passive or active action [116]. Examples of polymers with such an effect are chitosan [110], lysozyme [117] and bacteriocins [118]. Additionally, the antimicrobial properties of the polymer complexes are enhanced by introducing metals, e.g., silver [119], essential oils [120] or organic acids with a preservative effect [121] into their structure. Wu et al. developed a green process of anchoring nisin onto oxidized cellulose through a simple Schiff-based reaction [118]. As a result, antimicrobial active food packaging with an oxygen barrier property, water resistance and transmittance was obtained. Seydim et al. showed that whey protein foils enriched with oregano oil can be successfully used in the fight against *E. coli* [122]. Moreover, whey protein films with the addition of nisin and natamycin have been confirmed to exhibit high inhibition rates for the yeast and mould occurring naturally in Kasar cheese. In turn, bergamot and lemon oils were effective against *S. aureus* and *E. coli* [120].

#### 8.1.3. Active and Intelligent Packaging

Natural polymers used in packaging can perform various functions, depending on their physical and chemical properties. New generations of active and intelligent packaging are the future of the food packaging industry [123]. An important function of active packaging is to absorb moisture, which is the main cause of food spoilage. There are many compounds on the market that exhibit hygroscopic properties that include natural polymers such as sorbitol, xylitol, fructose and polyvinyl alcohol [124]. Cellulose and its derivatives also show good hygroscopic properties, which has been confirmed in many scientific studies [125]. In turn, Farooq et al., by utilizing a variety of different lignin morphologies, obtained cellulose nanofibril nanocomposite [126]. The obtained film exhibited complementary UV shielding and radical scavenging capability. Indumathi et al. proved that the combination of chitosan/cellulose acetate phthalate (CAP), incorporated with ZnO nanoparticles, allows one to obtain a structurally stable food packaging film appropriate for extending the shelf life of fresh black grapes in comparison with a commercial polyethylene cover. The water vapour transmission rate and oxygen transmission rate of such films were significantly lower in comparison with commercial polymeric films [127].

#### 8.1.4. Biosensors

Natural polymers are widely used as biosensors, substances that respond to an impulse from the external environment, in the form of a chemical or physical stimulus that causes a specific change in the properties of the material [128]. The way biosensors work is manifold due to their broad properties [129]. Used in food packaging, they monitor the food quality along the food chain, such as composition, storage conditions and bacterial growth [130]. An example would be anthocyanins extracted from natural fruits and vegetables as the source of pH-sensitive dyes. They produce different colour changes under acid and alkali environment so are largely used as colorimetric indicators in many foods’ intelligent packaging [118].

### 8.2. Non-Food Industry

Natural polymers such as rubbers and sluices are increasingly replacing synthetic materials due to their biocompatibility, low production cost, availability and non-toxicity. Due to their properties, they are products that are considered as alternative sources of raw materials for industrial applications [131].

**Figure 6:**
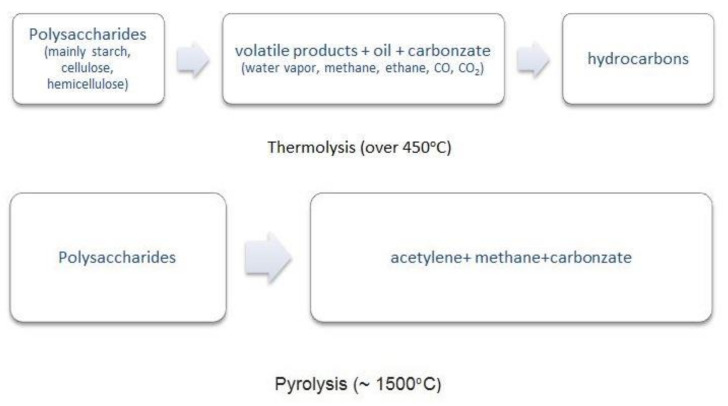
Fabrication of polysaccharides for non-food industry.

In the face of restrictions on food production imposed on Poland by the European Union, the use of arable crops for environmental engineering may be beneficial for domestic agriculture and industries cooperating with agriculture. The current state-of-the-art and known technologies make it possible to obtain, from plants and plant materials, saccharides and a series of polysaccharides as basic materials for the chemical industry [132] (Figure 6). The diagram shows the possibilities of non-food use of polysaccharides, where a valuable raw material can also be found, e.g., cellulose and hemicellulose.

Domestic polysaccharide resources can be used without deep processing, adapting them to different purposes through physical, physicochemical, chemical and enzymatic modification. Examples of such applications may be adhesives; microcapsules; absorbents; and additives for the production of pulp, paper, biodegradable materials and plasticizers, which are important in the new generation of ceramics [133,134,135,136,137].

Starches, irrespective of their botanical origin and their constituents, i.e., amylose and amylopectin, readily form Werner-type complexes when their gels are mixed with aqueous solutions of transition metal salts. This gives the possibility of its use as sorbents in the separation of metal ions and wastewater treatment [138].

Studies on the thermal degradation of starch grains and grains coordinated with metal ions have shown that the coordination of polysaccharides contained therein with the metal can control the yield of the resulting char and volatile products. Carbonizates are potential starting materials for the production of second-generation biofuels [139]. It was also found that thermal decomposition at a temperature much lower than that of a polysaccharide not coordinated with metal ions is possible [140,141,142,143].

Research is being conducted on the use of starch of various botanical origins (potato, corn, waxy maize, tapioca and amaranth) and cereals (barley, oats, wheat, triticale and rye) as soil stabilizers, drilling muds, metal ion collectors and a source of biofuels through degradation to synthetic gas and char [132].

Activated carbons derived from grains and cereal straw are widely used in environmental fields such as groundwater [144], volatile organic compound (VOC) control [145] and wastewater treatment [146]. Zhen Li et al. described many industrial residues and agricultural and forestry products, as well as other cheap resources that can be used to prepare char/carbon [147,148].

Farm straw is also a promising raw material for active carbon production, mainly due to its ability to avoid environmental pollution through combustion. Activated carbons based on rice [149], wheat [150], sesame [151] and maize straw [152] were obtained.

Coal from different sources shows different adsorption properties. This allows for versatile applications. The content of lignin, cellulose and inorganic substances are all raw materials from which active carbon is produced. The choice of pyrolysis temperature, carbonization temperature and the addition of chemicals affect the formation of pores [153,154] and properties such as polarization, hydrophobicity, acidity and the adsorption capacity of active carbon [135,136,155].

The method of obtaining activated carbons has a large impact on the adsorption of heavy metals. Many authors have shown that activated carbons can be used to remove organic dyes and antibiotics from wastewater [156,157,158].

Native polysaccharides containing proteins and fats can also be used to obtain biochar from them. Produced from crops (straw and seeds), biochar is an excellent system for the sorption of, not only metal ions, but also for the sorption of pharmaceuticals (beta-blockers, anti-inflammatory drugs, sulfonamides, caffeine). The tested crop-derived biochar has great potential for soil improvement and wastewater treatment. The biochar derived from cereals is a useful material for application in environmental protection purposes [135,136].

An important factor for consumers in determining the quality of a product is colour, which is one of the most important factors influencing the appearance of a product. New ways to use natural dyes as replacements for these synthetic dyes are being explored. Some of them are very sensitive. A good method of increasing the use of sensitive natural dyes is to encapsulate these dyes in colloidal particles by natural polymers, which may be carbohydrates, lipids or proteins. In recent years, encapsulation has been increasingly used for various purposes, including in non-food products. This technique improves the stability of sensitive natural dyes and offers the possibility of entrapping water-insoluble dyes for better application in an aqueous system [159].

The systems presented in this study can be directly applicable, not only as starting materials for the production of biofuels, but also in various fields of engineering and environmental protection. Systems have properties that enable the capture of relatively large volumes of water, creating a “clathrates”. These properties allow them to be used, inter alia, as collector metal ion binding substances, able to separate heavy metals from soil, as stabilizing substances (organic fertilizer) on embankments and on the waterfront of rivers, and as components of drilling fluids, causing, in addition to the lifting of excavated material, the improvement of lubrication holes and drill levelling disorders [137,138].

Biopolymer materials are gaining more and more significance in medicine and biotechnology. Due to the increase in the number of microorganisms resistant to antibiotics and other antimicrobial agents, new and cost-effective solutions are sought to overcome drug resistance. Khachatryan et al., through the green synthesis of silver nanoparticles using hyaluronan as a stabilizing matrix, obtained composites with bacteriostatic activity against *E. coli*, *Staphylococcus* spp. and *Bacillus* spp. [160]. Souza et al. also discussed polysaccharide-based materials resulting from physical processes and their biomedical use. These structures are created by combining charged polyelectrolytes in aqueous solutions without the use of toxic chemicals (cross-linkers). They examined main polysaccharides (glycosaminoglycans, marine polysaccharides and derivatives) containing ionizing groups in their structure as well as cellulose (neutral polysaccharide). They reviewed strategies including coacervation, ionotropic gelling, electrospinning, layer-by-layer coating, gelling of polymer mixtures, solvent evaporation and freeze-thaw methods. They focused on materials used to deliver growth factor (GF), scaffolds, antimicrobial coatings and wound dressings [161,162].

### 8.3. Future Prospective

The use of biodegradable natural polymers in the food and non-food industry is wide, and it is impossible to list all examples of their application here. The detailed knowledge of their properties broadens the possibilities of their use, which makes them more and more competitive in relation to plastics. It is undeniable that the subject of biodegradable materials obtained from renewable sources with specific properties is intensively researched. The research conducted in this area will have a huge impact on our future life. Their main goal is to reduce environmental pollution and stop the degradation of soil, air and water, consequently, improving the quality and safety of human life.

## 9. Conclusions

The gathered experience clearly demonstrates that the polysaccharide component of ternary complexes must possess an anionic character.

Ternary complexes create the foundations for a new generation of biodegradable engineering plastics (e.g., disposable tableware, packaging) and food (edible casings and packaging, microcapsules) plastics. Due to good mechanical properties, these novel plastics may fulfil many requirements of humanity as stable materials for everyday use. The composition of complexes and novel compounds may determine more ecological solutions. Other applications may be found, such as safe and edible toys for small children. The edible toys may be a solution, and prevent the swallowing of non-edible plastics by children. In addition, is important for future generations to replace many non-degradable plastics with ternary complexes from biopolymers.

## Data Availability

The data presented in this study are available on request from the corresponding author.
